# Neglected Tropical Diseases in the Catholic World

**DOI:** 10.1371/journal.pntd.0001132

**Published:** 2011-04-26

**Authors:** Peter J. Hotez

**Affiliations:** 1 Sabin Vaccine Institute, Washington, D.C., United States of America; 2 Department of Microbiology, Immunology, and Tropical Medicine, George Washington University Medical Center, Washington, D.C., United States of America

*Roughly one-quarter of the world's most common neglected tropical diseases and almost all of the cases of Chagas disease occur in the Catholic majority countries of Africa, Asia, and Latin America. This finding highlights new opportunities to lift the poorest Catholics in developing countries out of poverty.*

The neglected tropical diseases (NTDs) are the most common infections of the world's poorest people living in developing countries. Through their impact on child growth and development, pregnancy outcome, and worker productivity, the NTDs cause a massive global disease burden and have also been shown to promote poverty and economic underdevelopment [1]. In previous analyses, I have suggested that the NTDs are not evenly distributed throughout the developing countries of the tropics [2], [3]. Instead, approximately 30%–50% of the most common NTDs, such as intestinal helminth infections, schistosomiasis, and trachoma, occur in the nations that comprise the Organisation of the Islamic Conference, especially the poorest Islamic countries in Asia and Africa [2]. Another 20%–30% of these high prevalence NTDs are found among the poorest people who live in large middle-income countries such as India, China, Pakistan, and Iran. Despite their huge disparities of income and levels of poverty, ironically, these same countries also have tremendous scientific prowess, including the capacity to produce and maintain nuclear arsenals [3].

**Figure 1:**
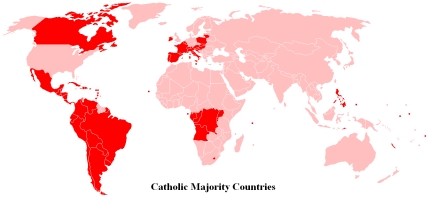
Catholic majority countries. From [30].

The next largest category of countries with a high prevalence of NTDs is countries with Catholic majorities. Listed in Table 1 (and shown in Figure 1) are the nations where most of the world's 1.1 billion Catholics live [4]. Only countries with Catholic majorities are included (in addition to Canada and Uganda, each with more than 40% of its population Catholic). Not included are large NTD disease-endemic nations such as India, Indonesia, Kenya, Nigeria, and Vietnam that contain sizeable Catholic minority populations. At least 5 million Catholics live in each of the 31 countries in Table 1, and together the 818 million Catholics living in these countries comprise three-quarters of the world's Catholic population. Seventeen of the countries are in the Latin American and Caribbean region, led by Brazil and Mexico (the most populous Catholic countries with 146 million and 123 million Catholics each, respectively), followed by the Philippines, as the only Asian country on the list (70 million), and the European nations of Italy and France. Four sub-Saharan African countries are listed in Table 1, including the Democratic Republic of Congo (DRC) with 30 million Catholics, Angola and Uganda (10–11 million each), and Burundi (5 million).

**Table 1:** NTDs in the Catholic World.

#^a^, ^b^	Country^a^, ^b^	Catholics^a^, ^b^	% Catholic^a^, ^b^	Cases of Ascariasis^c^	Cases of Trichuriasis^c^	Cases of Hookworm^c^	Cases of Schistosomiasis^d^	LF or Oncho Transmission +/–e	Chagas Transmission +/−f, g
1	Brazil	145 million	79%	42 million	19 million	32 million	2–7 million	+	+
2	Mexico	123 million	87%	9 million	18 million	1 million	—	+	+
3	Philippines	70 million	81%	41 million	38 million	18 million	<1 million	+	—
4	Italy	58 million	97%	—	—	—	—	—	—
5	France	44 million	76%	—	—	—	—	—	—
6	Colombia	38 million	86%	6 million	15 million	3 million	—	+	+
7	Spain	37 million	88%	—	—	—	—	−	—
8	Poland	35 million	94%	—	—	—	—	−	—
9	Argentina	34 million	89%	8 million	3 million	2 million	—	−	+
10	DRC	30 million	50%	23 million	26 million	31 million	15 million	+	—
11	Peru	28 million	88%	7 million	8 million	1.5 million	—	−	+
12	Venezuela	25 million	88%	7 million	9 million	1.5 million	—	+	+
13	Canada	13 million	44%	—	—	—	—	—	—
14	Ecuador	12 million	90%	5 million	2 million	1 million	—	+	+
15	Uganda	11 million	42%	4 million	3 million	9 million	5 million	+	—
16	Chile	11 million	71%	3 million	3 million	—	—	−	+
17	Guatemala	10 million	77%	8 million	9 million	3 million	—	+	+
18	Angola	10 million	50%	3 million	<1 million	11 million	6 million	+	−
19	Portugal	9 million	90%	—	—	—	—	−	—
20	Bolivia	8 million	85%	1 million	<1 million	1 million	—	−	+
21	Dom. Rep.	8 million	86%	<1 million	<1 million	<1million	<1 million	+	—
22	Belgium	8 million	76%	—	—	—	—	−	—
23	Haiti	7 million	65%	3 million	4 million	1 million	—	+	—
24	Cuba	6 million	50%	<1million	3 million	1 million	—	−	—
25	Hungary	6 million	58%	—	—	—	—	−	—
26	Honduras	6 million	79%	2 million	3 million	1 million	—	−	+
27	Austria	6 million	72%	—	—	—	—	−	—
28	El Salvador	5 million	76%	2 million	3 million	<1 million	—	−	+
29	Paraguay	5 million	92%	1 million	<1 million	3 million	—	−	+
30	Nicaragua	5 million	82%	<1 million	1 million	<1 million	—	−	+
31	Burundi	5 million	65%	1 million	1 million	2 million	1 million	+	—
	Totals for Catholic countries	818 million		176 million	168 million	123 million	29–34 million	13/31	14/31
	Worldwide	6.87 billion^g^		807 million	604 million	576 million	207 million		
	Catholics	12%		22%	28%	21%	14%–16%	ND	100%

a Catholic populations by country from http://www.catholic-hierarchy.org/country/sc1.html [4].

b Only the top 31 Catholic countries with more than 5 million Catholics and countries in which at least 50% of the population is Catholic are included (as well as Canada and Uganda, each with more than 40% Catholic population), which excludes India, Indonesia, Kenya, Nigeria, and Vietnam.

c From [6], [7].

d From [8], [9].

e From [10], [11].

f Chagas disease is found in every South American and Central American country listed [5].

g From [31].

While none of the eight European countries (or Canada) in Table 1 suffer from widespread NTDs, all of the Latin American nations listed, as well as the African Catholic countries and the Philippines, are highly endemic for such conditions. Together, these 22 nations comprise a significant percentage of the world's NTDs (Table 1), including almost 100% of the Chagas cases, which are found overwhelmingly in Latin America [5], and 21%–27% of the world's intestinal helminth infections, led by Brazil in Latin America, the Philippines in Asia, and DRC in sub-Saharan Africa [6], [7]. The 22 most populous NTD-endemic Catholic countries also account for 14%–16% of the world's 207 million cases of schistosomiasis [8], [9], and lymphatic filariasis (LF), onchocerciasis, or both of these infections are transmitted in 13 of the 22 nations [10], [11].

A critical policy implication of these findings is that the major Catholic charities and perhaps even the Catholic Church has a unique opportunity to promote NTD control in several of the 22 most endemic Catholic majority countries. Today, several Catholic charities are making significant contributions to global public health, including efforts to promote “deworming”, i.e., mass drug administration for human intestinal helminth infections. Catholic Relief Services has assisted poor and vulnerable populations for over 60 years [12], [13], with specific efforts to provide deworming treatments for school-aged children in Benin [14], Ghana [15], and presumably elsewhere in over 30 African countries where they work [16], while also operating community health programs in 26 countries that serve 3.5 million people [17]. Similarly, the Catholic Medical Mission Board, which was founded in 1928 by Dr. Paluel Flagg after a visit to Haiti to help leprosy patients, has provided support to health care programs in developing countries since 1966 and today distributes hundreds of millions of dollars worth of medicines annually [18]. Many local archdioceses, including the Roman Catholic Archdiocese of Manila through its Caritas Manila program, for example, also provide deworming treatments [19]. Of interest is the finding that many Catholic organizations operate in countries both with and without Catholic majority populations, recognizing health as a fundamental right for people of all denominations [16], [17].

The important work of the major Catholic charities and the Church could ultimately be expanded to support national programs of NTD control and elimination. Currently, the United States Agency for International Development (USAID) is supporting integrated control of the seven most common NTDs through low-cost packages of essential medicines in 14 countries including four of the 22 listed in Table 1, i.e., DRC, Haiti, the Philippines, and Uganda [20], while the British Department for International Development (DFID) is also supporting additional African countries [21]; both agencies are planning to expand their NTD control activities in the coming years. Additional countries, including Burundi, are being supported through Legatum, Geneva Global, and the Global Network for NTDs [22].

In an earlier Editorial, I pointed out that it is unreasonable to expect the United States and the United Kingdom to shoulder the entire burden of global NTD control and that we must look to other European countries, the Gulf Cooperation Council, and even some emerging economies for development assistance on this front [23]. But, in addition, the major Catholic charities and the Catholic Church could re-organize some of their advocacy and resource mobilization programs to combat NTDs among the world's poorest Catholics, including national control programs in the 17 countries not currently supported by USAID, DFID, or the Global Network for NTDs. Efforts could include partnerships with the major Catholic majority endemic countries in Latin America, Africa, and the Philippines. Through mass administration with a package of preventive chemotherapy drugs costing as little as US$0.50 annually [24], [25], such global action could directly benefit the 100–200 million Catholics suffering today from intestinal helminth infections, the roughly 30 million with schistosomiasis, and tens of millions with LF and/or onchocerciasis. Today, the so-called rapid impact packages of NTD control medicines are considered among the lowest cost interventions in all of global health, and one of the most cost-effective [1], [25]. Partnerships with the Catholic Church or charities could also help to reduce the global disease burden of Chagas disease, which, because it is found almost exclusively found in the countries of Latin America, is largely a disease of Catholics, as well as selected indigenous groups [5].

The Catholic Church has also supported scientific research and development in tropical medicine, beginning with the origin of the antimalarial drug, quinine, from “Jesuit's bark” or “Jesuit's powder” [26], [27]. Today, Rev. Thomas Streit, CSC, from the University of Notre Dame, is leading efforts to eliminate LF through mass drug administration in Haiti [28]. Science-based institutions such as the Pontifical Academy of Sciences founded by Pope Pius XI in the 1930s [29] could also provide important guidance for NTD research and new product development.

Expanded global public health efforts through programs of national control, stepped-up advocacy, and resource mobilization efforts by the major Catholic charities, nongovernmental development organizations affiliated with the Church, and of course the Holy See, could ultimately lead to reductions of one-quarter of the world's intestinal helminth infections, 15% of the world's schistosomiasis, and much of the world's Chagas disease. At a modest cost of between US$50 and US$100 million annually, such global action would establish an amazing legacy for health and economic development by and for the global Catholic community.
